# Dual Biologic Therapy for the Treatment of Pediatric Inflammatory Bowel Disease: A Review of the Literature

**DOI:** 10.3390/jcm11072004

**Published:** 2022-04-03

**Authors:** Magdalena Wlazło, Jarosław Kierkuś

**Affiliations:** Department of Gastroenterology, Hepatology, Feeding Disorders and Pediatrics, The Children’s Memorial Health Institute, 04-730 Warsaw, Poland; j.kierkus@ipczd.pl

**Keywords:** pediatric inflammatory bowel disease, combination biologics, colitis ulcerosa, Crohn’s disease

## Abstract

Background: pediatric patients with inflammatory bowel diseases (IBD) who qualify for biological therapy represent a group of severely ill patients. They have never been successful with conventional medication. Biologic medications in monotherapy are frequently used in the disease course, however they result in a 1-year remission, which can be maintained in approximately 40% of IBD patients. Method: the present study aims to summarize the review of literature data on the use of therapy with a combination of two biological and small molecule drugs, anti-TNF (infliximab, adalimumab), vedolizumab and ustekinumab, as well as Janus kinase inhibitors (tofacitinib). The risks associated with the use of dual biological therapy and potential adverse effects are particularly important. The literature data was reviewed using the following terms: “use of combination biologic in paediatric IBD”, “combination biologics”, and “dual biologic for treatment of Inflammatory Bowel Disease”. Conclusion: the use of dual biological therapy is a new therapeutic option. In pediatric IBD, combining the different mechanisms of action of the two biological drugs seems to be safe and effective. Anti-TNF drugs with vedolizumab or ustekinumab may be a particularly beneficial combination. Nevertheless, the clarification and justification of potential advantages of combined biological therapies in further studies, such as randomized control trials, are needed.

## 1. Introduction

The prevalence of inflammatory bowel diseases (IBDs), a term mainly used to refer to Crohn’s disease (CD) and ulcerative colitis (UC), continues to increase, especially in industrialized countries. It is noticeable among adult patients, but in pediatric patients most of all. [1] This progressive trend is associated with unhealthy diets and environments, which affect the intestinal microbiome. The pathogenesis of Crohn’s disease and ulcerative colitis involves an inappropriate response of the immune system to commensal microbiota in genetically susceptible patients [2]. Genetic predisposition may be of great importance in the development of IBD, especially in the youngest patients [3]. 

Pediatric inflammatory bowel diseases are characterized by a diverse presentation of the disease and variable clinical courses. The therapeutic aims are to induce and maintain remission. Despite the many therapeutic options available, patients often fail to achieve clinical and endoscopic remission. The established biological therapy in pediatric IBD has focused on the use of tumor necrosis factor (TNF) medications [4] Anti-TNF-α therapies are approved in induction and maintenance therapy for the treatment of moderate-to-severe pediatric IBD [5,6]. Biologic agents are now commonly used in the disease course, but clinical remission is achievable in only 40–60% of patients receiving anti-TNF monotherapy [5,6]. Other groups of biological drugs, such as vedolizumab, an α4β7 integrin antagonist, and ustekinumab, which binds to the common p40 chain of interleukin IL-12 and IL-23, are frequently used off-label in children as second-line biologic agents. Patients who did not respond satisfactorily to treatment after induction with anti-TNF are 27% less likely to benefit from second-line biologics [7,8]. According to the limited pediatric data, patients who previously have lost response to one of the biological therapies achieved 38.6–58% remission at week 52 of treatment with ustekinumab [9]. In 2016, Conrad et al. [10] published a retrospective study that describes the adverse events and clinical response to vedolizumab in pediatric IBD. A clinical response in week 22 was observed in 11/19 (57.9%) patients with UC or IBD unclassified (IBD-U) and 9/15 (60.0%) patients with CD. In a retrospective review with 16 VEO-IBD (very early onset disease) patients (4 CD patients and 12 CU patients) [11] who received vedolizumab, a clinical response after induction therapy was noticed in 9/16 (56.3%) children. In Crohn’s disease (CD) and ulcerative colitis (UC), most of the experience relied on a combination biological therapy with immunomodulators [12], but in the pediatric inflammatory bowel disease group of patients, no information is available regarding the effects of a long-term combination of biological therapies. This article aims to summarize the currently available literature on the use of dual biologics in pediatric IBD. The published literature in this article includes retrospective studies, case series, and case reports. This article presents the efficacy and safety of combination therapies and evaluates the potential benefits of combination biological therapies in pediatric IBD.

## 2. Materials and Methods

We found 5 publications reporting the use of a combination of biological agents (TNF antagonists, VDZ or UST) in pediatric inflammatory bowel disease (Table 1). The PubMed and MEDLIN databases were searched, using the terms “use of combination biologic in paediatric IBD”, “combination biologics”, and “dual biologic for treatment of Inflammatory Bowel Disease”.

## 3. Results

### 3.1. Case Report and Case Series

In 2019, Bass J. and Goyal A. published a case report of a 14-year-old patient with a clinically severe form of CD, sacroiliitis and growth failure [13]. He was diagnosed with inflammatory bowel disease at nine years of age. Induction treatment with systemic steroids and azathioprine was ineffective. The patient was qualified for biological treatment with anti-tumor necrosis factor (TNF) drugs, initially with infliximab for 1 year, and then with adalimumab for 1.5 years with a suboptimal improvement of the patient’s condition. He required prolonged administration of vancomycin in connection with recurrent Clostridioides difficile infections. Due to the lack of clinical remission during the use of anti-TNF drugs, therapy was changed. He was qualified for treatment with vedolizumab, a systemic steroid and again switched to azathioprine. Despite the patient’s clinical improvement, laboratory tests showed persistent anemia, elevated markers of inflammation, and severe growth deficiency. An endoscopic evaluation revealed active inflammation in the large intestine and terminal ileum with progressive disease in the distal colon. Mesalazine and oral budesonide included in the treatment, were proved unsuccessful. The intervals for the administration of vedolizumab doses were shortened to four weeks. Treatment with azathioprine was withdrawn due to its lack of efficacy. Considering the significant body-weight deficiencies, enteral nutrition was started for three months. During treatment, the patient reported pain in the lower limbs and hips. A rheumatological evaluation was performed. The diagnosis of sacroiliitis was based on a physical examination and diagnostic imaging. It was decided to use a combination of two biological drugs. Adalimumab was added to the therapy with vedolizumab. In addition, due to the increased symptoms of rheumatological disease, the patient was administered oral methotrexate. During nine months of treatment, no side effects were reported. The patient showed a clinical response with a significant reduction in inflammatory markers and recovered from anemia. His body weight increased by 25 kg in 12 months of treatment.

In one of the first pediatric studies, 8 patients aged 14–17.5 years (4 CD patients and 4 CU patients) received combined biological therapy with infliximab and vedolizumab [14,15]. All patients had endoscopically and histopathologically confirmed active intestinal inflammations before the escalation of therapy. To confirm clinical remission, a pediatric disease activity scale (PUCAI—Pediatric Ulcerative Colitis Activity Index or PCDAI—Pediatric Crohn’s Disease Activity Index), markers of inflammation and fecal calprotectin levels were used. Clinical remission was achieved in 4 (50%) patients (3 CU). Despite dual biological therapy, 4 (50%) patients required colectomy (3 CD, 1 UC). In addition, five patients developed psoriasis classified as a treatment complication during therapy with infliximab. With the addition of ustekinumab to treatment in this group of patients, it resulted in a significant clinical improvement with relief of skin symptoms in 3 out of 5 (60%) patients. In one of them, partial improvement in the underlying disease and skin lesions was achieved. One patient did not respond to treatment. No serious adverse effects were observed during dual biological therapy.

The most commonly used dual biological therapy consisted of an anti-TNF agent and a newer biologic but very limited primary evidence, such as case series [16], suggested that the use of ustekinumab and vedolizumab may be effective for refractory Crohn’s disease with fistula. Howard et al. [16] published a case of a patient with severe, refractory CD treated with conventional IBD pharmacotherapy and biological therapy (infliximab, adalimumab and vedolizumab). She underwent a subtotal colectomy. During the operation, an ileo-sigmoid anastomosis with a loop ileostomy was performed. A few months later, a recto-vaginal fistula with an anal ulcer developed. Induction treatment with ustekinumab with ciprofloxacin was ineffective. Despite clinical improvement, her disease was in the active phase. It was decided to add vedolizumab to the treatment with ustekinumab. After three weeks, the vagino-rectal fistula was completely healed. In the pediatric Crohn’s disease activity index (PCDAI), the patient scored 5 compared with 40 at the beginning of the therapy. The patient reported much better wellbeing, but she did not achieve endoscopic remission. Another patient was a 20-year-old man with a diagnosis of severe oral and perianal Crohn’s disease. At the age of 12, he underwent hemicolectomy with an ileostomy. He was treated with methotrexate, courses of antibiotics, and biological drugs, such as infliximab, adalimumab and vedolizumab. He initially had a good response to ustekinumab treatment, but developed deep ulcers distal and proximal to the anastomosis after attempting to restore the integrity of the gastrointestinal tract. The addition of budesonide and cholestyramine to therapy did not improve the patient’s clinical condition. He complained of severe abdominal pain and diarrhea. Budesonide was excluded from treatment. Ustekinumab was added to treatment with vedolizumab. The patient achieved clinical and endoscopic remission after 20 weeks of using double biological therapy.

The third patient was a 17-year-old female with Crohn’s disease from the age of 8 years. She had a loss of response to treatment with azathioprine, infliximab, adalimumab with methotrexate, and an incomplete response to vedolizumab and Crohn’s Disease Exclusion Diet. While on monotherapy with ustekinumab, the patient developed fever, hypoalbuminemia, and anemia. High inflammatory markers and the value of calprotectin above 5.000 (mg/g) were worth noting. Endoscopy revealed severe pancolitis. Additionally, the patient had ileal disease with a fistula in the terminal ileum. For this reason, she was hospitalized and received antibiotics. A few weeks before the elective surgery, vedolizumab was added to the therapy with ustekinumab. She had a short course of steroids added as a bridge to vedolizumab. After 4 weeks, she obtained steroid-free clinical remission with albumin increased to normal, decreased CRP and PCDAI from 55 to 2.5. Comparable to the previous patient, no endoscopic remission was observed.

### 3.2. Retrospective Study

The first retrospective study, from 2020, includes 9 patients with CD, aged 8 to 19 years [17]. The study aims were to review patients who received dual biologic therapy for at least four weeks. The effectiveness and safety of the treatment were assessed. A total of 6 (67%) patients were treated with a combination of infliximab/adalimumab with vedolizumab, and 2 (22%) patients were given ustekinumab and vedolizumab for refractory disease. One patient received infliximab with anakinra (a drug from the group of human interleukin-1 receptor antagonists). According to the authors, 4 (44%) patients achieved remission; two patients (22%) did not respond to the therapy; one (11%) had an improvement in growth; And two patients (22%) had a partial response. The publication does not include information on the criteria for remission in the publication. One patient had a staphylococcal skin infection during dual biological therapy. Dolinger et al. [18] analyzed the effectiveness and safety of combined biological therapy. Their study is the first to combine dual biological therapy with tofacitinib (a drug from the group of Janus kinase inhibitors—JAK) in a pediatric treatment population. The study comprises 16 patients with severe IBD (9 patients with UC/unclassified enteritis and 7 patients with CD), who failed at least 2 biological therapies and were enrolled in the study. The patients were divided as follows: 9 (56%) were included in the therapy with vedolizumab and tofacitinib, 4 (25%) patients with ustekinumab and vedolizumab and the other 3 (19%) with ustekinumab and tofacitinib. Of the analyzed group, 12 (75%) patients (7 UC/IBD-U and 5 CD) achieved steroid-free clinical remission at 6 months (remission was defined as Crohn’s disease activity in children with a CDAI ≤ 12.5 or Mayo < 2). The median time to clinical remission without steroids was 88 days. Three patients (19%) discontinued therapy due to ineffectiveness. One of them required surgery, the other two patients needed to switch to another dual biological therapy regimen. Importantly, one serious adverse event of septic arthritis occurred after two months of treatment with vedolizumab and tofacitinib.

## 4. Discussion

This review compares five publications with the use of a combination of biological agents (TNF antagonists, VDZ or US and tofacitinib) in pediatric inflammatory bowel disease. This article represents a review of the literature that show the effectiveness of dual biological therapy in active pediatric IBD. In a study involving the highest number of pediatric patients, 12 (75%) patients achieved steroid-free clinical remission at 6 months. One patient required surgery, the other two patients needed to switch their therapy [18]. In a subsequent retrospective study [17], 4 (44%) patients achieved clinical remission. Olbjørn reported clinical remission in 9 (70%) of the 13 patients; 4 patients required a colectomy (3 CD, 1 UC) [14]. Howard et al. [16] published a case of a patients with severe CD treated with vedolizumab and ustekinumab. In this study, all patients achieved clinical remission, but endoscopic remission was not observed for all patients. It seems that this combination of biological drugs (VDZ + UST) may be particularly useful in the treatment of patients with fistulas, leading to the closure of a fistula and obtaining continuity after the takedown of a stoma. These reports are also confirmed by publications in the adult population of patients suffering from inflammatory bowel diseases [19,20,21]. The authors emphasize that they decided to use such a combination of two biological drugs because of a better safety profile than in the case of using anti-TNF drugs. This is a very important aspect, especially in the group of pediatric patients [22,23]. The treatments with combinations of biological medications seems to be a safe and well-tolerated option for pediatric IBD. In the first retrospective study [20], nine patients with CD were included, and one patient had a staphylococcal skin infection during dual biological therapy. In 2021, Dolinger published a study that reported the occurrence of one serious adverse event of septic arthritis after two months of treatment with vedolizumab and tofacitinib [21]. The economic aspect of treatment with dual biological therapy is also important. The costs of such a form of therapy are higher than conventional treatment with the use of immunomodulators; however, the failure of clinical remission during standard pharmacological treatment implies an increased risk of hospitalization, surgery and, consequently, reduced quality of life [24]. Moreover, the onset of activity of some biological drugs, such as vedolizumab, may even develop several months after the initiation of therapy, much later than the activity of anti-TNF drugs [25]. In patients who partially improved on vedolizumab monotherapy, the addition of an anti-TNF agent may have a bridging effect until the expected effect of vedolizumab is achieved. In this way, we avoid the use of high doses of corticosteroids and the associated, often irreversible, side effects [26].

## 5. Conclusions

The use of dual biological therapy is a new opportunity for patients with limited treatment options remaining. It presents a chance to achieve clinical and endoscopic remission in patients resistant to standard pharmacological treatment. Based on the available data, it seems that the use of two drugs with a different mechanism of action is a safe and effective form of therapy. The combination of anti-TNF drugs with vedolizumab or ustekinumab seems to be of particular benefit. Randomized prospective clinical trials with more patients included in the study are necessary to assess the efficacy and safety of dual biology versus monotherapy.

## Figures and Tables

**Table 1:** A Review of the literature on dual biologics for the treatment of pediatric IBD.

Study	Year	Study Types	Therapy	Number of Patients	Duration of Combination Therapy (Average)	Efficacy	Adverse Events
Bass et al.	2019	CR	VDZ + ADA	1	9 mth	Clinical response with a significant reduction in inflamatory markers.	None.
Olbjořrn et al.	2020	CS	IFX + UST IFX + VDZ	5 8	36 mth 10 mth	Clinical remission in 9 of the 13 patients.	5 patients had severe paradoxical psoriasis on maintenance therapy with IFX.
Howard et al.	2021	CS	VDZ + UST	3	7 mth	Clinical remision; no endoscopic remission was observed.	None.
Goyal et al.	2020	RS	Anty-TNF + VDZ VDZ + UST IFX + anakinra	6 2 1	11 mth	4 (44%) patients achieved clinical remission;	One patient had a staphylococcal skin infection.
Dolinger et al.	2021	RS	VDZ + tofacitnib VDZ + UST UST+ tofacitinb	9 4 3	6 mth	12 (75%) patients achieved steroid-free clinical remission at 6 months; One of them required surgery, the other 2 patients needed to switch to therapy.	One serious adverse event of septic arthritis occurred after 2 months of treatment with vedolizumab and tofacitinib.

ADA, adalimumab; CR, case report; CS, case series; IFX, infliximab; mth, month; TNF, tumor necrosis factor; UST, ustekinumab; and VDZ, vedolizumab.

## Data Availability

Data may be obtained from the corresponding author upon submission of a written request.
